# Prognostic value of the triglyceride-glucose (TyG) index for renal function progression in patients with CKD stages 3–4

**DOI:** 10.3389/fnut.2026.1744275

**Published:** 2026-04-28

**Authors:** Fan Zhu, Huihui Mao, Wenyuan Gan, Xingruo Zeng, Licong Su, Wenli Chen

**Affiliations:** 1 Department of Nephrology, The Central Hospital of Wuhan, Tongji Medical College, Huazhong University of Science and Technology, Wuhan, China; 2 Division of Nephrology, National Clinical Research Center for Kidney Disease, State Key Laboratory of Organ Failure Research, Nanfang Hospital, Southern Medical University, Guangzhou, China; 3 Department of Nutrition, The Central Hospital of Wuhan, Tongji Medical College, Huazhong University of Science and Technology, Wuhan, China

**Keywords:** chronic kidney disease, insulin resistance, nonlinear relationship, renal function progression, triglyceride-glucose index

## Abstract

**Background:**

The progression of chronic kidney disease (CKD) is a major public health issue. Insulin resistance (IR) is a key mechanism, but simple biomarkers to predict progression in patients with established CKD stages 3–4 are needed. The Triglyceride-glucose (TyG) index is a reliable surrogate for IR. We aimed to investigate the longitudinal association between the baseline TyG index and the risk of renal function progression in patients with CKD stages 3–4.

**Methods:**

This multicenter retrospective cohort study included 53,607 patients with CKD stages 3–4 from the China Renal Data System (CRDS) between 2005 and 2022. The baseline TyG index was calculated, and participants were divided into quartiles. The primary outcome was renal function progression. We used multivariate Cox proportional hazards models, restricted cubic splines (RCS), subgroup analyses, and competing-risk models to assess the association.

**Results:**

During a median follow-up of 31.18 months, 19,619 (36.6%) patients experienced renal function progression. After full multivariate adjustment, each 1-unit increase in the continuous TyG index was associated with a 10% higher risk of renal function progression (Hazard Ratio [HR] 1.10, 95% CI 1.07–1.12). Compared to the second quartile (Q2, reference), the highest quartile (Q4) had a significantly increased risk (HR 1.17, 95% CI 1.12–1.22). The RCS analysis revealed a non-linear, U-shaped association between the TyG index and renal function progression, with the lowest risk (nadir) at a TyG index of approximately 8.6–8.8. This U-shaped association was consistent across subgroup analyses and robust in sensitivity analyses, including a competing-risk model treating all-cause death as the competing event.

**Conclusion:**

The TyG index is an independent predictor of renal function progression in patients with CKD stages 3–4, demonstrating a U-shaped relationship. As an inexpensive and readily available marker, the TyG index may serve as a valuable tool for early risk stratification in this high-risk population.

## Introduction

Chronic Kidney Disease (CKD) represents a significant global health challenge, characterized by a progressive loss of kidney function over time (1, 2). The burden of CKD is substantial, leading to increased risks of end-stage renal disease (ESRD), cardiovascular complications, and premature mortality (3). Particularly, individuals with CKD stages 3–4, defined by a glomerular filtration rate (GFR) of 15–59 mL/min/1.73m^2^, are at a critical juncture where interventions to slow progression and mitigate complications are paramount (4). Identifying modifiable risk factors and early biomarkers associated with accelerated renal function decline in this population is therefore of utmost importance for improving patient outcomes (5).

Insulin resistance (IR) has emerged as a key pathophysiological mechanism implicated in the development and progression of CKD (6, 7). IR can contribute to glomerular hyperfiltration, renal inflammation, fibrosis, and endothelial dysfunction, all of which are hallmarks of CKD progression (8). While the hyperinsulinemic-euglycemic clamp is the gold standard for measuring IR, its complexity and cost limit its use in routine clinical practice and large-scale epidemiological studies (9). This has spurred the search for simpler, more accessible surrogate markers of IR.

The Triglyceride-glucose (TyG) index, calculated as Ln [fasting triglycerides (mg/dL) × fasting glucose (mg/dL)/2], has gained considerable attention as a cost-effective and reliable indicator of IR [13, 14]. A growing body of evidence supports the association of an elevated TyG index with various metabolic disorders, including type 2 diabetes mellitus, non-alcoholic fatty liver disease, and cardiovascular diseases (10, 11). More recently, research has begun to explore the link between the TyG index and kidney dysfunction. Studies have demonstrated that a higher TyG index is associated with an increased prevalence and incidence of CKD in diverse populations (12). Furthermore, the TyG index has been linked to markers of renal damage, such as albuminuria and reduced eGFR (13).

However, while the association between the TyG index and the presence of CKD is increasingly recognized, its specific role in predicting the progression of renal dysfunction in patients already diagnosed with moderate to severe CKD (stages 3–4) warrants further investigation. Patients in these stages often exhibit a complex interplay of metabolic abnormalities, and understanding the contribution of IR, as reflected by the TyG index, to their subsequent renal decline is crucial (14). Some studies have suggested a link between the TyG index and faster GFR decline or increased risk of ESRD in broader CKD populations or specific subgroups like diabetic nephropathy (15), but dedicated research focusing on the nuanced progression within CKD stages 3–4 is less abundant.

Therefore, this study aims to investigate the longitudinal association between baseline TyG index and the rate of renal function progression in a well-characterized cohort of patients with CKD stages 3–4. Elucidating this relationship could provide valuable insights into the pathogenic role of insulin resistance in this specific patient population and may help identify individuals at higher risk for accelerated kidney function loss. Ultimately, the TyG index could serve as a readily available and inexpensive biomarker to aid in risk stratification and guide targeted therapeutic strategies aimed at mitigating insulin resistance and preserving renal function in patients with established CKD.

### Study population

**Figure 1:**
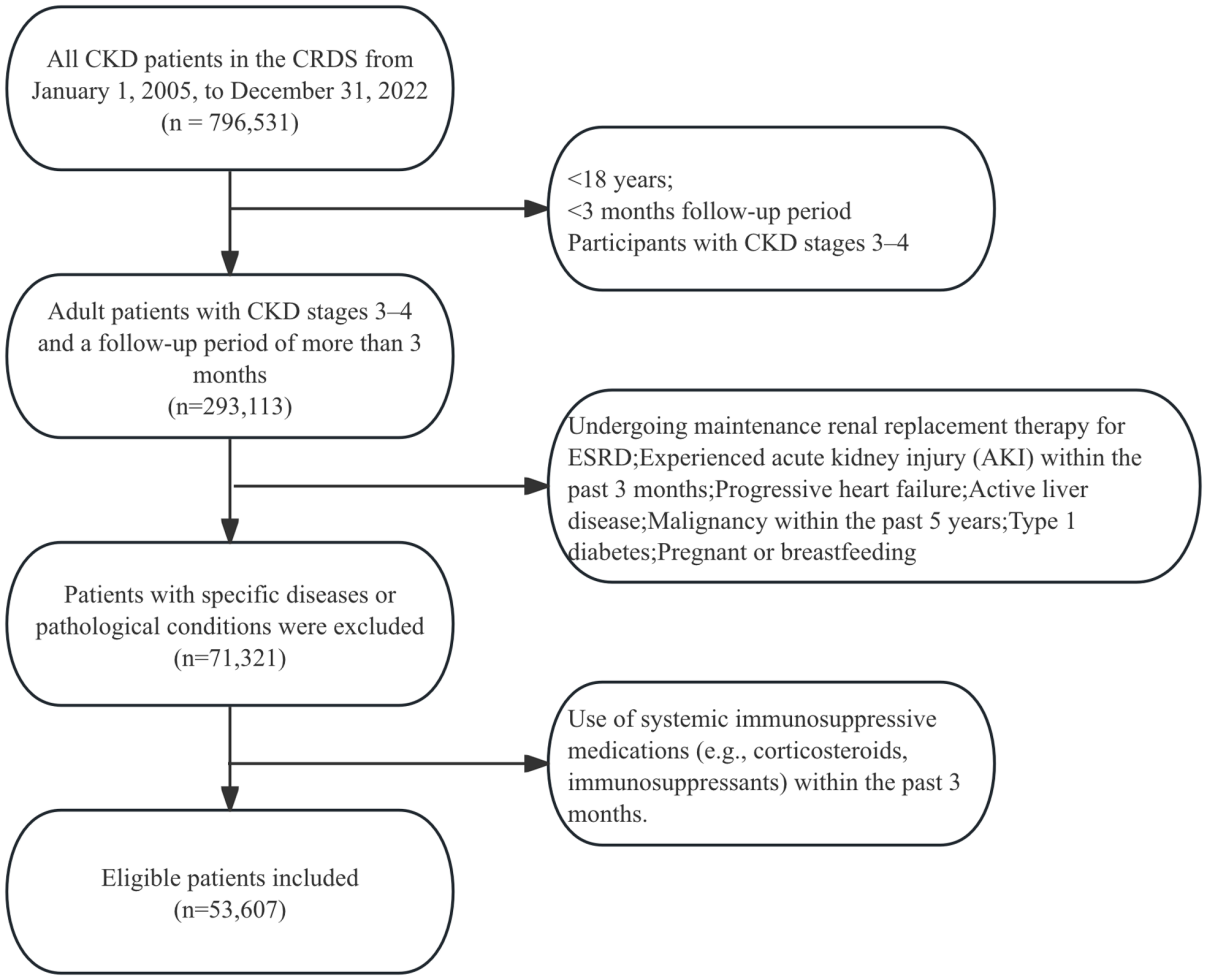
Patient screening flowchart.

Our research is a multicenter retrospective cohort study utilizing the China Renal Data System (CRDS), targeting all individuals diagnosed with CKD during their hospital stays or outpatient visits between January 1, 2005, and December 31, 2022. As shown in Figure 1, within the period, a total of 796,531patients were considered for CKD diagnosis in the CRDS. After applying the inclusion and exclusion criteria, 53,607 patients were included in the final analysis.

#### Inclusion criteria

(1) Participants must be 18 years of age or older at the time of enrollment, with a follow-up period exceeding 3 months.
(2) Participants must have a documented diagnosis of CKD, defined as evidence of kidney damage or a decreased glomerular filtration rate (GFR) for three or more months.
(3) Participants must be in CKD stage 3 or 4 at the time of screening, based on their estimated Glomerular Filtration Rate (eGFR). The eGFR will be calculated using the Chronic Kidney Disease Epidemiology Collaboration (CKD-EPI) equation. Stage 3: eGFR between 30 and 59 mL/min/1.73 m^2^; Stage 4: eGFR between 15 and 29 mL/min/1.73 m^2^.

#### Exclusion criteria

(1) End-Stage Kidney Disease (ESKD): History of or current treatment with any form of renal replacement therapy (hemodialysis, peritoneal dialysis, or kidney transplantation) at baseline; eGFR < 15 mL/min/1.73 m^2^ (CKD stage 5).
(2) Acute Kidney Injury (AKI): A history of AKI within the 3 months prior to enrollment, defined as a rapid decline in renal function. This is to ensure that the baseline eGFR is stable and representative of the participant’s chronic condition.
(3) Specific Medical Conditions: Known diagnosis of type 1 diabetes mellitus; Active malignancy or a history of malignancy within the past 5 years; Severe, decompensated liver disease (e.g., cirrhosis with a Child-Pugh score of B or C); Advanced heart failure (New York Heart Association [NYHA] Class IV).
(4) Current use of medications known to significantly alter glucose or lipid metabolism, such as high-dose systemic corticosteroids, if the dosage has not been stable for at least 3 months prior to enrollment.
(5) Participants who are pregnant, planning to become pregnant during the study period, or are breastfeeding.

### Study outcomes and endpoints

Follow-up for each participant commenced at the baseline visit and continued until the occurrence of a primary or secondary endpoint, or the end of the study period (December 31, 2022), whichever came first.

### Primary outcome

The primary outcome was a composite endpoint of renal function progression. This was defined as the first occurrence of any of the following events:

A sustained decline in estimated Glomer Filtration Rate (eGFR) of 40% or more from the baseline measurement.

Progression to End-Stage Renal Disease (ESRD), defined as a sustained eGFR less than 15 mL/min/1.73 m^2^.

The initiation of maintenance renal replacement therapy (RRT), including chronic hemodialysis, peritoneal dialysis, or receiving a kidney transplant.

Serial eGFR values were calculated using the Chronic Kidney Disease Epidemiology Collaboration (CKD-EPI) equation. A sustained decline in eGFR was defined as a value that was confirmed by at least one subsequent measurement 90 days or more apart, to minimize misclassification due to acute kidney injury (AKI). Data on RRT initiation and kidney transplantation were obtained from the China Renal Data System (CRDS) registry and hospital records.

### Secondary outcome

The secondary outcome for this study was all-cause mortality. Data on mortality were ascertained from hospital discharge records and vital status information linked within the CRDS.

### Endpoint adjudication and competing risk

All outcome events were tracked through the CRDS database and electronic medical records. For the analysis of the primary renal outcome, all-cause death occurring before the composite renal endpoint was treated as a competing event.

### Ethical approval and informed consent

This study was conducted in accordance with relevant guidelines and regulations, including the principles of the Declaration of Helsinki. Ethical approval was granted by The Central Hospital of Wuhan Ethics Committee (Approval No. WHZXKYL2022-112), and all experimental protocols were approved by the same committee. Informed consent was obtained from all subjects and/or their legal guardians prior to data collection.

### Informed consent

All participants must be capable of understanding the study and willing to provide written informed consent before any study-related procedures are performed.

### Data availability

Participants must have available baseline laboratory data for the calculation of the TyG index, including fasting triglycerides and fasting plasma glucose, and complete medical records.

### Statistical analysis

Patients were divided into quartiles based on TyG index values. Depending on the distribution type, continuous variables were displayed as mean ± standard deviation or median and interquartile range (25th and 75th percentile). Differences between groups were assessed using ANOVA or the Kruskal–Wallis H test, depending on whether the distribution was normal. Categorical variables were expressed as numbers and proportions, and comparisons were made using either the Chi-square test or Fisher’s exact test, as appropriate. The Kaplan–Meier (KM) curves were used to describe survival across different TyG quartiles, and the log-rank test was used to determine group differences. Univariate and multivariate Cox regression models were employed to explore the relationship between the TyG index and renal function progression. Model 1 was unadjusted for TyG, whereas Model 2 included age and gender as covariates; ethnic adjustment was not performed as the cohort consisted of a homogeneous population. Model 3 was fully adjusted (excluding TG and glucose) and included variables with a *p*-value of less than 0.10 in the univariate Cox regression analysis (for renal function progression occurrence). Missing values were handled using multiple imputation by chained equations (MICE). Variables with more than 10% missing data were excluded from analysis, while those with less than 10% missing data were imputed using MICE. Restricted cubic spline plots (with five knots) were used to assess the nonlinear relationship between the TyG index and primary outcomes. We fitted Fine–Gray subdistribution hazard models for renal function progression, treating all-cause death prior to renal function progression as the competing event. We modeled TyG with restricted cubic splines using five knots to balance flexibility and parsimony in this large cohort. Knots were placed at the 5th, 27.5th, 50th, 72.5th, and 95th percentiles and kept identical across Models 1–3. Likelihood-ratio tests supported the spline specification over a linear term, and a 4-knot alternative yielded similar inferences.

Two-sided *p*-values less than 0.05 were considered statistically significant. Statistical analyses were performed using R software, version 4.2.1. Data analysis employed the R packages mice, dplyr, survminer, ggplot2, survival, tidyr, foreign, and rms.

## Results

### Baseline characteristics

As shown in Table 1, we included 53,607 patients with CKD stages 3–4, categorized by TyG quartiles (Q1: 7.98–8.34; Q2: 8.57–8.78; Q3: 9.00–9.24; Q4: 9.57–10.22) with 13,403/13,400/13,403/13,401 participants, respectively. Overall follow-up was 31.18 ± 28.64 months (*p* < 0.001 across quartiles). Men comprised 61.2% of the cohort and median age was 65.65 years [54.88–73.48] (both *p* < 0.001). Hypertension and diabetes were present in 50.7 and 34.5% (both *p* < 0.001). Use of RAS inhibitors, lipid-lowering agents, insulin, and diuretics was 41.8%, 41.2%, 23.1%, and 28.0%, respectively (all *p* < 0.001). The mean Charlson Comorbidity Index was 4.27 ± 1.63 (*p* < 0.001).

**Table 1:** Baseline clinical characteristics of patients according to tertiles of triglyceride-glucose index.

TyG index	Overall [8.46, 9.39]	Q1 [7.98, 8.34]	Q2 [8.57, 8.78]	Q3 [9.00, 9.24]	Q4 [9.57, 10.22]	*p*
N	53,607	13,403	13,400	13,403	13,401	
Follow-up duration	31.18 ± 28.64	31.97 ± 28.54	31.71 ± 29.30	31.47 ± 28.75	29.54 ± 27.88	<0.001
Sex, (Male, %)	32,793 (61.2)	8,802 (65.7)	8,257 (61.6)	8,032 (59.9)	7,702 (57.5)	<0.001
Age, year	65.65 [54.88, 73.48]	67.78 [56.51, 74.84]	66.44 [55.40, 74.10]	65.00 [54.49, 72.89]	63.55 [53.69, 71.60]	<0.001
RASi (%)	22,408 (41.8)	5,079 (37.9)	5,364 (40.0)	5,672 (42.3)	6,293 (47.0)	<0.001
Hypertension (%)	27,197 (50.7)	6,287 (46.9)	6,583 (49.1)	6,942 (51.8)	7,385 (55.1)	<0.001
Diabetes Mellites (%)	18,513 (34.5)	2,902 (21.7)	3,410 (25.4)	4,593 (34.3)	7,608 (56.8)	<0.001
Diuretics (%)	15,015 (28.0)	3,851 (28.7)	3,536 (26.4)	3,662 (27.3)	3,966 (29.6)	<0.001
LLA (%)	22,111 (41.2)	4,659 (34.8)	5,013 (37.4)	5,611 (41.9)	6,828 (51.0)	<0.001
Insulin (%)	12,389 (23.1)	1918 (14.3)	2087 (15.6)	2,856 (21.3)	5,528 (41.3)	<0.001
BMI, kg/m^2^	24.22 [24.21, 24.97]	24.22 [22.99, 24.97]	24.22 [24.11, 24.97]	24.22 [24.22, 24.97]	24.22 [24.22, 24.97]	<0.001
SBP, mmHg	147.24 (75.78)	146.84 (79.65)	147.23 (78.64)	147.95 (76.34)	146.93 (67.93)	0.623
DBP, mmHg	82.13 (12.45)	81.76 (13.02)	82.09 (12.78)	82.67 (13.15)	81.95 (11.89)	0.587
Scr, mmol/l	133.90 [113.34, 173.90]	132.00 [113.00, 170.00]	133.00 [113.00, 172.00]	134.00 [113.00, 174.93]	136.00 [114.70, 177.70]	<0.001
Bun, mmol/l	8.33 [6.56, 11.00]	8.20 [6.43, 10.83]	8.15 [6.48, 10.71]	8.30 [6.52, 10.95]	8.70 [6.82, 11.50]	<0.001
Hb, g/l	124.29 (22.76)	120.72 (23.01)	124.21 (22.62)	125.74 (22.50)	126.50 (22.49)	<0.001
ACR, mg/g	791.71 (2003.29)	550.39 (1561.73)	661.39 (1688.10)	811.67 (1914.67)	1143.41 (2626.75)	<0.001
Hs-CRP	18.30 [5.00, 186.68]	18.30 [5.00, 164.00]	18.30 [5.00, 201.18]	18.30 [5.00, 168.70]	18.30 [5.00, 201.25]	0.986
LDL, mmol/l	2.72 [2.07, 3.46]	2.38 [1.80, 3.02]	2.72 [2.10, 3.40]	2.89 [2.23, 3.65]	2.92 [2.20, 3.78]	<0.001
TC, mmol/l	4.62 [3.80, 5.57]	4.11 [3.38, 4.91]	4.51 [3.74, 5.35]	4.79 [3.97, 5.73]	5.16 [4.25, 6.26]	<0.001
HDL, mmol/l	1.07 [0.87, 1.32]	1.17 [0.94, 1.45]	1.10 [0.90, 1.34]	1.04 [0.85, 1.26]	0.99 [0.81, 1.21]	<0.001
Tg, mmol/l	1.53 [1.08, 2.25]	0.89 [0.73, 1.06]	1.37 [1.17, 1.57]	1.91 [1.57, 2.26]	2.97 [2.19, 4.05]	<0.001
Alb, g/l	39.21 [35.00, 43.00]	38.40 [34.50, 42.00]	39.50 [35.40, 43.00]	39.91 [35.60, 43.60]	39.20 [34.40, 43.10]	<0.001
Glucose, mmol/l	5.58 [4.85, 7.18]	4.89 [4.39, 5.44]	5.31 [4.79, 6.10]	5.83 [5.10, 7.16]	8.19 [6.00, 11.98]	<0.001
UA, mmol/l	440.00 [363.00, 526.00]	427.00 [351.00, 513.00]	437.00 [361.00, 522.00]	446.00 [367.10, 530.00]	449.00 [372.00, 536.00]	<0.001
K, mmol/l	4.12 [3.78, 4.50]	4.12 [3.78, 4.50]	4.11 [3.78, 4.48]	4.12 [3.79, 4.50]	4.13 [3.77, 4.52]	0.099
Na, mmol/l	140.44 [138.00, 142.40]	140.80 [138.26, 142.80]	141.00 [138.60, 143.00]	140.80 [138.20, 142.64]	139.64 [137.00, 142.00]	<0.001
Cl, mmol/l	104.80 [101.84, 107.50]	105.00 [102.10, 108.00]	105.00 [102.00, 107.80]	104.91 [102.00, 107.50]	103.90 [100.60, 106.80]	<0.001
P, mmol/l	1.14 [0.99, 1.30]	1.12 [0.98, 1.28]	1.13 [0.98, 1.29]	1.14 [0.99, 1.30]	1.16 [1.00, 1.32]	<0.001
Ca, mmol/l	2.26 [2.15, 2.37]	2.24 [2.13, 2.34]	2.26 [2.16, 2.37]	2.28 [2.16, 2.38]	2.27 [2.16, 2.38]	<0.001
MACE (%)	12,498 (23.3)	2,988 (22.3)	3,010 (22.5)	3,078 (23.0)	3,422 (25.5)	<0.001
Charlson. Score	4.27 (1.63)	4.25 (1.59)	4.22 (1.55)	4.23 (1.63)	4.38 (1.75)	<0.001
Renal function progression (%)	19,619 (36.6)	4,715 (35.2)	4,492 (33.5)	4,831 (36.0)	5,581 (41.6)	<0.001
Death (%)	4,802 (9.0)	1,429 (10.7)	1,009 (7.5)	1,118 (8.3)	1,246 (9.3)	<0.001

Data presentation: data are presented as mean (SD), *n* (%), or median [25th - 75th percentile].

BMI, body mass index; Hb, hemoglobin; Scr, serum creatinine; BUN, blood urea nitrogen; UA, uric acid; Alb: albumin.

eGFR: estimated glomerular filtration rate, hs-CRP: high-sensitivity C-reactive protein, TG: triglycerides, HDL-C: high-density lipoprotein cholesterol.

LDL-C: low-density lipoprotein cholesterol, RASi: renin-angiotensin system inhibitors, LLA: lipid-lowering agents.

Blood pressure showed no between-quartile differences (SBP *p* = 0.623; DBP *p* = 0.587). Renal parameters (serum creatinine, blood urea nitrogen) and ACR differed across quartiles (all *p* < 0.001). Albumin differed (*p* < 0.001), whereas hs-CRP did not (*p* = 0.986). The metabolic profile (fasting glucose, triglycerides, total cholesterol, LDL-C, HDL-C) differed across quartiles (all *p* < 0.001). For electrolytes, potassium showed no difference (*p* = 0.099), while sodium, chloride, phosphate, and calcium differed (all *p* < 0.001). Event distribution at baseline: MACE 23.3%, renal function progression 36.6%, and all-cause death 9.0%; each differed across TyG quartiles (all *p* < 0.001).

### Renal survival across TyG index quartiles

**Figure 2:**
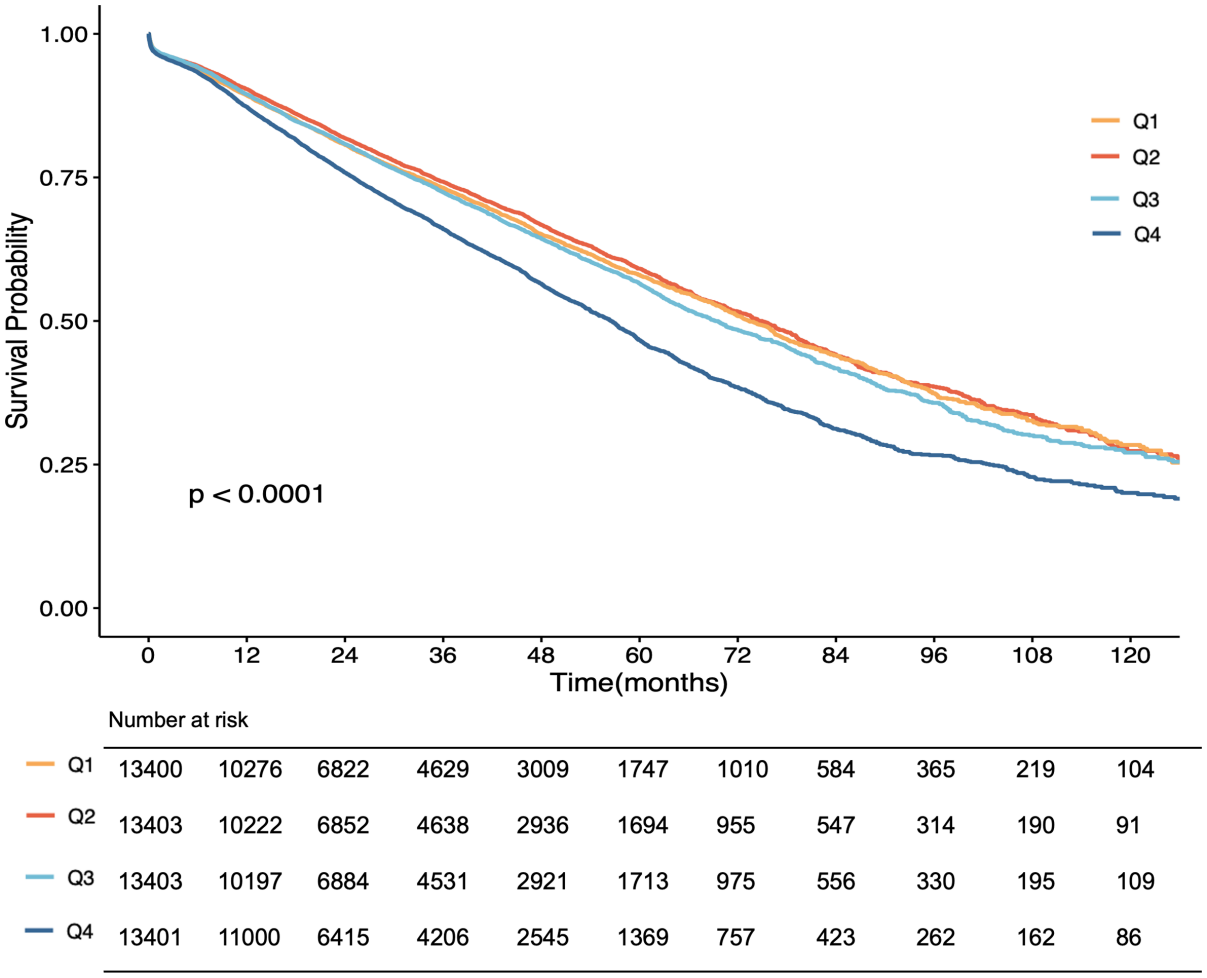
Survival outcomes by different TyG groups: renal function progression according to quartiles of TyG index.

Figure 2 shows participants in the highest TyG quartile (Q4) exhibited a significantly lower renal survival probability, indicating a higher risk of renal function progression. The survival curves for Q1 to Q3 were relatively close, while Q4 consistently remained separated and inferior across the duration of follow-up.

### TyG index and risk of renal function progression across models

Table 2 presents the association between TyG index and renal function progression under different models In Cox models, higher TyG was associated with greater risk of renal function progression. Per 1-unit increase in TyG, the hazard ratio (HR) was 1.17 (95% CI, 1.15–1.19) unadjusted, 1.15 (1.13–1.17) adjusted for age/sex, and 1.10 (1.07–1.12) fully adjusted (all *p* < 0.001). Using quartiles with Q2 as reference, Q4 showed a consistent association across models—HR 1.39 (1.34–1.43), 1.36 (1.31–1.40), and 1.17 (1.12–1.22) in Models 1–3 (all *p* < 0.001). Q3 was significant before full adjustment (Model 1: HR 1.08 [1.04–1.13], *p* = 0.023; Model 2: HR 1.07 [1.02–1.11], *p* = 0.004) but not after full adjustment (HR 1.02 [0.98–1.07], *p* = 0.374). Q1 was not significant in the fully adjusted model (HR 1.01 [0.96–1.05], *p* = 0.738).

**Table 2:** The association between various TyG index and renal function progression.

TyG	Model1	*p*-value	Model2	*p*-value	Model3	*p*-value
	HR (95% CI)		HR (95% CI)		HR (95% CI)	
Continues	1.17 (1.15–1.19)	**<0.001**	1.15 (1.13–1.17)	**<0.001**	1.10 (1.07–1.12)	**<0.001**
Quartiles						
Q1	1.05 (1.01–1.09)	**0.036**	1.05 (1.01–1.10)	**0.028**	1.01 (0.96–1.05)	0.738
Q2	Ref	Ref	Ref	Ref	Ref	Ref
Q3	1.08 (1.04–1.13)	**0.023**	1.07 (1.02–1.11)	**0.004**	1.02 (0.98–1.07)	0.374
Q4	1.39 (1.34–1.43)	**0.022**	1.36 (1.31–1.40)	**<0.001**	1.17 (1.12–1.22)	**<0.001**

Model 1: Unadjusted.

Model 2: Adjusted for age and sex.

Model 3: adjusted for age, sex, diastolic blood pressure, BMI, comorbidities (hypertension, diabetes mellitus), laboratory test results (Hb, Alb, UA, eGFR, Scr, BUN, ACR, hs-CRP, LDL-C, HDL-C, TC, K, Na, Cl, P, Ca, Proteinuria), and medications (Insulin, RAS inhibitors, lipid-lowering agents [LLA], diuretics). Values in bold indicate statistical significance with a *p*-value < 0.05.

### Nonlinear association between TyG and renal function progression: RCS (models 1–3)

**Figure 3:**
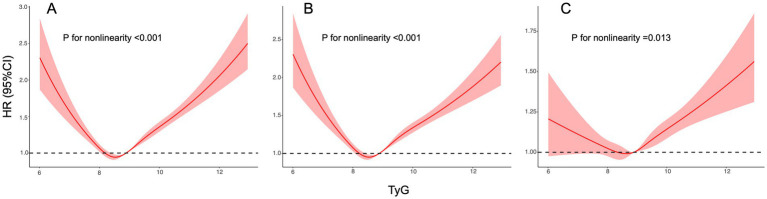
Restricted cubic spline curves of the association between TyG and renal function progression. **(A)** Unadjusted model. **(B)** Adjusted for age and sex. **(C)** Fully adjusted model. Hazard ratios are indicated by solid red lines and 95% CIs are indicated by shaded areas. CI, confidence interval; TyG, triglyceride-glucose index.

Five-knot RCS curves (Figure 3, panels A–C for Models 1–3) showed a non-linear association between TyG and renal function progression in all models, with a common nadir around TyG ≈ 8.6–8.8 (HR ≈ 0.95–1.00). Above ~9.0, the hazard rose consistently: at TyG ≈ 10 the HR was ~1.20 in Models 1–2 and ~1.10 in Model 3; at TyG ≈ 12.5 the HR reached ~2.5 (Model 1), ~2.2 (Model 2), and ~1.6 (Model 3). At low TyG (~6.0), the HRs were ~2.2 (Model 1), ~2.3 (Model 2), and ~1.2 (Model 3). These dose–response patterns mirror the quartile findings, with risk clearly exceeding 1.0 for values corresponding to Q4 and higher, and with magnitude attenuated after multivariable adjustment while the shape remained unchanged.

### Subgroup analyses

**Figure 4:**
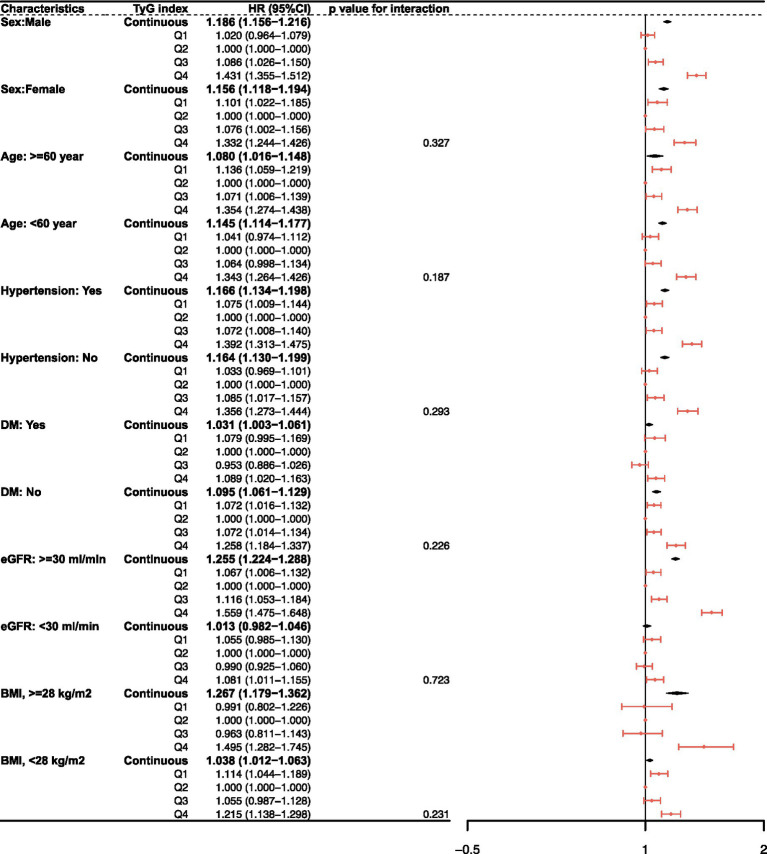
Subgroup analysis for TyG and renal function progression.

Subgroup analyses showed association between TyG index and risk of renal function progression across all strata (Table 3; Figure 4). Compared with the second quartile (Q2, reference group), both the lowest (Q1) and highest (Q4) TyG quartiles were associated with an increased risk of renal function progression in males (Q1: HR 1.186, 95% CI 1.156–1.216, *p* < 0.001; Q4: HR 1.431, 95% CI 1.355–1.512, *p* < 0.001) and females (Q1: HR 1.156, 95% CI 1.118–1.194, *p* < 0.001; Q4: HR 1.332, 95% CI 1.244–1.426, *p* < 0.001). Similar U-shaped trends were observed in both age subgroups (≥60 and <60 years), hypertension and diabetes status, baseline eGFR, and BMI categories. In the eGFR ≥30 mL/min group, Q1 (HR 1.255, 95% CI 1.224–1.288, *p* < 0.001) and Q4 (HR 1.559, 95% CI 1.475–1.648, *p* < 0.001) showed higher risks than Q2. In the BMI ≥ 28 kg/m^2^ group, the risks in Q1 (HR 1.267, 95% CI 1.179–1.362, *p* < 0.001) and Q4 (HR 1.495, 95% CI 1.282–1.745, *p* < 0.001) were also higher than in Q2. There were no statistically significant interactions between TyG index and any subgroup variable (all P for interaction >0.05) indicating that the association between higher TyG and renal function progression was broadly stable across strata, as illustrated in the forest plot.

**Table 3:** Subgroup analysis of the association between TyG index and renal function progression.

Characteristics	TyG index					P-interaction
	Continuous	Q1	Q2	Q3	Q4	
Sex						0.263
Male	1.186 (1.156–1.216)***	1.020 (0.964–1.079)	Ref	1.086 (1.026–1.150)**	1.431 (1.355–1.512)***	
Female	1.156 (1.118–1.194)***	1.100 (1.022–1.185)*	Ref	1.076 (1.002–1.156)*	1.332 (1.244–1.426)***	
Age, years						0.101
≥60	1.080 (1.016–1.148)*	1.136 (1.059–1.219)***	Ref	1.071 (1.006–1.139)*	1.354 (1.274–1.438)***	
<60	1.145 (1.114–1.177)***	1.041 (0.974–1.112)	Ref	1.064 (0.998–1.134)	1.343 (1.264–1.426)***	
Hypertension						0.144
Yes	1.166 (1.134–1.198)***	1.075 (1.009–1.144)*	Ref	1.072 (1.008–1.140)*	1.392 (1.313–1.475)***	
No	1.164 (1.130–1.199)***	1.033 (0.969–1.101)	Ref	1.085 (1.017–1.157)*	1.356 (1.273–1.444)***	
DM						0.205
Yes	1.031 (1.003–1.061)*	1.079 (0.995–1.169)	Ref	0.953 (0.886–1.026)	1.089 (1.020–1.163)*	
No	1.095 (1.061–1.129)***	1.072 (1.016–1.132)*	Ref	1.072 (1.014–1.134)*	1.258 (1.184–1.337)***	
eGFR, ml/min						0.613
≥30	1.255 (1.224–1.288)***	1.067 (1.006–1.132)*	Ref	1.116 (1.053–1.184)***	1.559 (1.475–1.648)***	
<30	1.013 (0.982–1.046)	1.055 (0.985–1.130)	Ref	0.990 (0.925–1.060)	1.081 (1.011–1.155)*	
BMI, kg/m^2^						0.112
≥28	1.267 (1.179–1.362)***	0.991 (0.802–1.226)	Ref	0.963 (0.811–1.143)	1.495 (1.282–1.745)***	
<28	1.038 (1.012–1.063)**	1.114 (1.044–1.189)**	Ref	1.055 (0.987–1.128)	1.215 (1.138–1.298)***	

DM, diabetes mellitus; eGFR, estimated glomerular filtration rate; BMI, body mass index.

Statistical significance: *p < 0.05, **p < 0.01, ***p < 0.001.

To further assess whether this association was driven by baseline clinical heterogeneity, we examined the consistency of the TyG-renal progression relationship across major cardiometabolic strata. The overall pattern remained stable in patients with and without hypertension, with and without diabetes, and across different baseline eGFR categories, with no evidence of significant interaction. These findings suggest that the prognostic relevance of TyG is not restricted to a specific subgroup but is broadly applicable across common CKD risk profiles.

### Sensitivity analysis

#### Complete-case vs. multiple imputation

Estimates were highly consistent between datasets (Supplementary Table S1). In complete-case analysis (*n* = 47,950; 17,545 events), Q4 vs. Q2 was associated with higher risk of renal function progression (HR 1.16, 95% CI 1.11–1.22), whereas Q1 (1.02, 0.98–1.06) and Q3 (1.03, 0.99–1.08) were not significant; P for trend <0.001. Results were similar after multiple imputation (*n* = 53,607; 19,619 events): Q4 1.17 (1.12–1.22), Q1 1.01 (0.97–1.05), Q3 1.02 (0.98–1.07); P for trend <0.001. For TyG as a continuous variable, the HR per 1-unit increase was 1.09 (1.06–1.12) in complete cases and 1.10 (1.07–1.12) with imputation (both *p* < 0.001). These findings indicate that handling of missing data did not materially affect the association between TyG and renal function progression.

#### In time-segmented analyses

In Supplementary Table S2, higher TyG (per 1-unit) was associated with renal function progression overall (HR 1.017, 95% CI 1.015–1.020; *p* < 0.001) and within each follow-up window: 0–1 year (1.063, 1.027–1.100; *p* < 0.001), ≤2 years (1.085, 1.058–1.113; *p* < 0.001), and >2 years (1.208, 1.163–1.254; *p* < 0.001). Using quartiles with Q2 as reference, overall estimates were: Q1 HR 1.049 (1.003–1.097; *p* < 0.05), Q3 HR 1.081 (1.034–1.131; *p* < 0.001), and Q4 HR 1.386 (1.328–1.448; *p* < 0.001). By follow-up segments (Q2 as reference): Q4 showed a consistent association across all windows (HR 1.23–1.28; all *p* < 0.001). Q3 was significant only within ≤2 years (HR 1.09; *p* < 0.01) but not at 0–1 year or >2 years. Q1 showed modest elevations across windows (HR 1.07–1.11; *p* ≤ 0.05–0.01).

#### RCS by follow-up duration

The restricted cubic spline (RCS) curves for each follow-up segment (0–1 year, ≤2 years, >2 years) revealed a non-linear relationship between TyG and renal function progression, consistent with the overall analysis (Supplementary Figure S1). Specifically, for each follow-up duration, the hazard ratio (HR) remained stable at lower TyG levels, with a clear increase in HR for TyG values above approximately 8.5, which corresponds to the HR seen in the Q4 group. In the 0–1 year segment, the HR increased slightly above TyG ≈ 8.5, and the trend was more pronounced in the ≤2 years and >2 years segments, with HRs reaching approximately 1.5–2.0 at higher TyG values, similar to the trend observed in the quartile analysis.

#### Competing-risk analysis

In Fine–Gray models treating all-cause death prior to renal function progression as the competing event (adjusted as Model 3, Table 4), the association remained: Q4 vs. Q2 showed a higher subdistribution hazard (sHR 1.16, 95% CI 1.11–1.21; *p* < 0.001), whereas Q1 (sHR 1.03, 0.99–1.07; *p* = 0.12) and Q3 (sHR 1.02, 0.98–1.06; *p* = 0.28) were not significant. These findings are consistent with the fully adjusted Cox results, indicating robustness of the Q4 effect under competing-risk modeling.

**Table 4:** Association between TyG quartiles and renal function progression using Fine–Gray competing risk model (all-cause death as competing event).

TyG quartile	Subdistribution HR (95% CI)	*p*-value
Q1	1.03 (0.99–1.07)	0.12
Q2 (reference)	1.00 (reference)	—
Q3	1.02 (0.98–1.06)	0.28
Q4	1.16 (1.11–1.21)	<0.001

Adjusted as in Model 3, Q2 is the reference.

## Discussion

This study was a large-scale, multicenter retrospective cohort study based on the China Renal Data System (CRDS), enrolling 53,607 patients with CKD stages 3–4. Our main finding indicates that the baseline TyG index is independently associated with the risk of renal function progression. After full multivariate adjustment, each one-unit increase in the TyG index was associated with a 10% increase in the risk of renal function progression (HR 1.10, 95% CI 1.07–1.12). Compared to patients in the second quartile (Q2) of the TyG index, those in the highest quartile (Q4) had a significantly increased risk of renal function progression by 17% (HR 1.17, 95% CI 1.12–1.22). Furthermore, restricted cubic spline (RCS) analysis revealed a non-linear, U-shaped association between the TyG index and the risk of renal function progression, with the nadir (lowest risk) occurring at a TyG index of approximately 8.6–8.8. This association remained robust across various subgroup analyses and sensitivity analyses, including a competing-risk model.

Compared with previous studies, our findings extend the current evidence by demonstrating a robust U-shaped association between the TyG index and renal function progression specifically in patients with CKD stages 3–4. While most prior studies have focused on the detrimental effects of elevated TyG levels, relatively few have explored the potential risks associated with low TyG levels in advanced CKD populations. This distinction highlights the unique metabolic characteristics of patients with moderate-to-advanced CKD.

Insulin resistance (IR) is considered one of the core pathophysiological mechanisms in the development and progression of CKD (6–8). The TyG index, as a simple and reliable surrogate marker for IR (9, 10), has seen its relationship with kidney disease become a research hotspot in recent years. The findings of this study are largely consistent with the trends in previous literature.

First, numerous cross-sectional and cohort studies have confirmed that an elevated TyG index is associated with both the prevalence of CKD and an increased risk of new-onset CKD (12, 13). For example, the cohort study and meta-analysis by Ren et al. (12) showed that a high TyG index is an independent risk factor for developing CKD in the general population. Several other recent large-scale prospective studies have drawn similar conclusions (12). This study further confirms that among individuals already diagnosed with moderate-to-severe CKD (stages 3–4), an elevated TyG index also predicts poorer renal outcomes.

Second, studies focusing on specific populations (such as those with type 2 diabetes or hypertension) also support the predictive value of the TyG index for renal function progression (15–18). A 2024 study published in Diabetes Care noted that among patients with type 2 diabetes and CKD, an elevated TyG index was significantly associated with rapid eGFR decline and risk of progression to ESRD (16). The unique contribution of our study lies in its larger cohort size and its specific focus on the CKD 3–4 stage, providing strong evidence for the TyG index as a prognostic biomarker in this population.

The most notable finding of this study is the U-shaped association between the TyG index and renal function progression. The RCS curve (Figure 3) clearly shows that not only is a high TyG index (>9.0) associated with increased risk, but a very low TyG index (Q1 group, <8.34) also shows a trend toward increased risk. Although the HR for the Q1 group did not reach statistical significance after multivariate adjustment (HR 1.01, *p* = 0.738), this trend was observed in multiple subgroup analyses (as shown in Table 3) and at the low-value end of the RCS curve.

The mechanism by which a high TyG index (Q4) increases risk is clear, primarily attributable to severe IR (19). IR leads to nephron damage and fibrosis through multiple pathways, including glomerular hyperfiltration, activation of the RAAS system, promotion of inflammation and oxidative stress, and renal lipotoxicity (8, 20, 21).

However, the “U-shaped” curve phenomenon, where a low TyG index (Q1) is associated with poor prognosis, warrants more discussion. This finding suggests that a very low TyG index may not represent ideal metabolic health, especially in the CKD 3–4 population, which often suffers from chronic wasting. We speculate this may be related to the “reverse epidemiology” phenomenon (22). Patients with CKD stages 3–4 often present with varying degrees of protein-energy wasting (PEW) and a state of micro-inflammation (23). Extremely low levels of triglycerides and glucose (components of the TyG index) may be markers of malnutrition, sarcopenia, and poor overall health [26]. In this context, the state of malnutrition and wasting itself is a stronger predictor of renal function progression and mortality than IR (24). Therefore, the U-shaped curve of the TyG index may reflect two different pathological states: the high end represents the harm of “metabolic excess” and IR, while the low end may represent the risk of “nutritional depletion” and PEW (25).

The results of this study support and expand upon two primary theoretical models:

IR and Renal Lipotoxicity Model (High TyG Index): As mentioned, a high TyG index is a marker of IR. According to theoretical models established by DeFronzo (6) and Artunc et al. (8), among others, IR and hyperinsulinemia can damage the kidneys through hemodynamic (e.g., increased sodium retention, sympathetic nervous system activation) and non-hemodynamic pathways (e.g., promoting cell proliferation, inflammatory cytokine release). Furthermore, the “Renal Lipotoxicity” model posits that excessive circulating free fatty acids and triglycerides (characteristic of a high TyG index) can be ectopically deposited in the kidneys (especially in renal tubular epithelial cells and podocytes), inducing oxidative stress, endoplasmic reticulum stress, and apoptosis, ultimately leading to tubulointerstitial fibrosis and glomerulosclerosis (26, 27). The significantly high risk in our Q4 group supports this pathological model.

Malnutrition-Inflammation-Wasting Model (Low TyG Index): The low-value end of the U-shaped curve discovered in our study aligns with the “Malnutrition-Inflammation-Wasting Syndrome” (MIWS) or PEW model in chronic disease (23, 28). In patients with CKD stages 3–4, decreased appetite, metabolic acidosis, and persistent low-grade inflammation lead to increased body catabolism (29, 30). A low TyG index (reflecting low energy substrates) may be a biomarker of this wasting state. This model suggests that in this condition, the body lacks the necessary energy and protein reserves for repair and homeostasis, making the kidneys more vulnerable to secondary insults and thus accelerating functional loss (31, 32).

This study also has several important strengths. First, it was based on a large multicenter CKD stages 3–4 cohort, which enhanced the statistical power and generalizability within routine clinical settings. Second, we evaluated the TyG index, a simple, inexpensive, and readily available metabolic marker, which increases the potential clinical applicability of our findings. Third, the robustness of the results was supported by multiple analytical approaches, including multivariable adjustment, subgroup analyses, sensitivity analyses, restricted cubic spline modeling, and competing-risk analysis.

## Limitations

Despite the advantages of a large sample size and detailed data, this study has several limitations:

First, because of the retrospective observational nature of this study, the association between the TyG index and renal function progression should not be interpreted as causal. Although we adjusted for major clinical characteristics and baseline medication use, residual confounding from unmeasured or incompletely captured variables may still have influenced the observed results.

Second, although major clinical variables and medication use were adjusted for, detailed information on medication dosage, adherence, and longitudinal treatment changes was not available, which may introduce residual confounding. In addition, lifestyle factors such as smoking and alcohol consumption were not consistently recorded in the database and could not be included in the analysis.

Third, the TyG index was measured only once at baseline. Changes in the TyG index over time (e.g., due to medication or disease progression) could impact prognosis, and a single measurement cannot capture this dynamic.

Fourth, the TyG index is ultimately a surrogate marker for IR, not the gold standard (such as the hyperinsulinemic-euglycemic clamp). Although the TyG has been widely validated, its accuracy in representing IR in the specific population of CKD 3–4, which is affected by complications like malnutrition, may be compromised.

Fifth, this study cohort consists mainly of a Chinese population. Whether the results can be generalized to CKD 3–4 patients of other ethnicities or regions requires further verification, as different ethnicities may have variations in IR presentation and lipid metabolism (33, 34).

Despite these limitations, the consistency of our findings across multiple analytical approaches, including subgroup, sensitivity, and competing-risk analyses, supports the robustness of the observed association.

## Conclusion

In summary, this large-scale retrospective study demonstrates that the TyG index is an independent predictor of renal function progression in patients with CKD stages 3–4, and a U-shaped association exists between them.

This finding has important clinical implications. As an inexpensive and routinely available marker, the TyG index can be easily integrated into clinical practice for risk stratification in patients with CKD stages 3–4. Importantly, the observed U-shaped relationship suggests that both metabolic excess (high TyG) and potential undernutrition or protein-energy wasting (low TyG) should be considered in patient management. Therefore, the TyG index may provide a dual clinical signal to guide both metabolic optimization and nutritional assessment in routine nephrology care.

## Data Availability

The raw data will be made available by the corresponding authors upon reasonable request, ensuring compliance with the ethical and privacy guidelines of the China Renal Data System (CRDS).
