# Supplementary figures and images for: Prognostic value of the triglyceride-glucose (TyG) index for renal function progression in patients with CKD stages 3–4
Source: Front Nutr. 2026 Apr 28;13:1744275. doi: 10.3389/fnut.2026.1744275 (PMC13161084; doi:10.3389/fnut.2026.1744275)

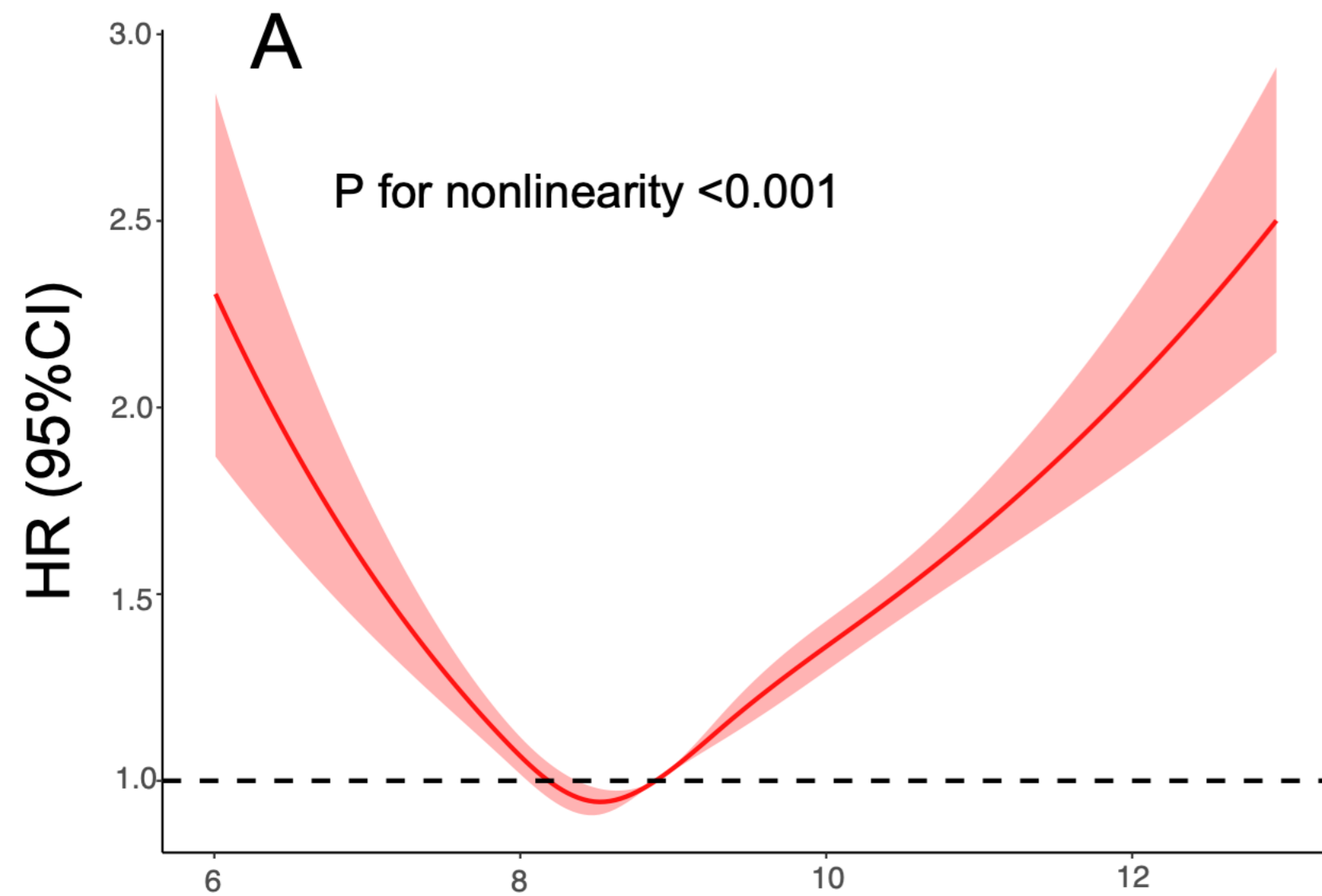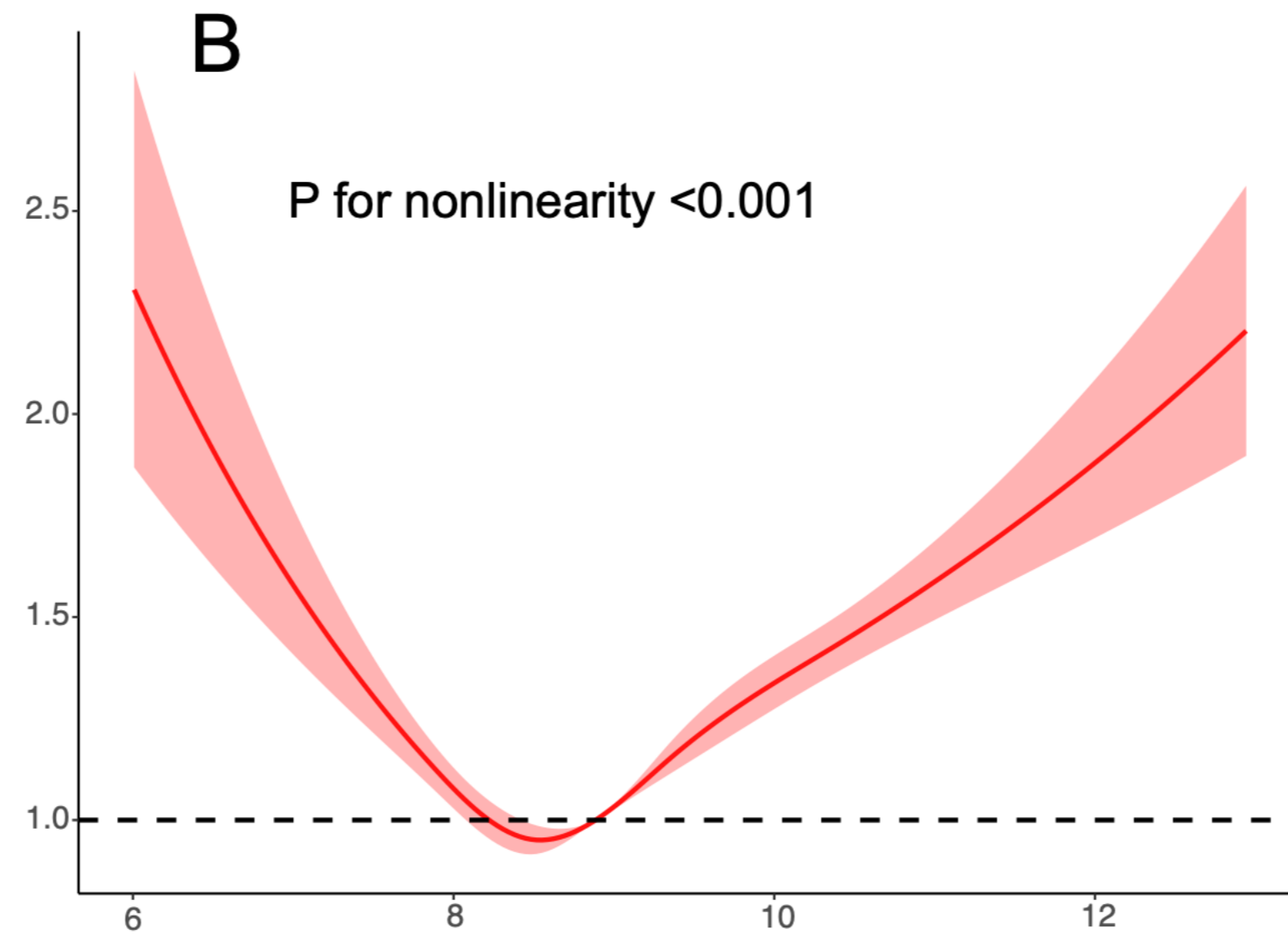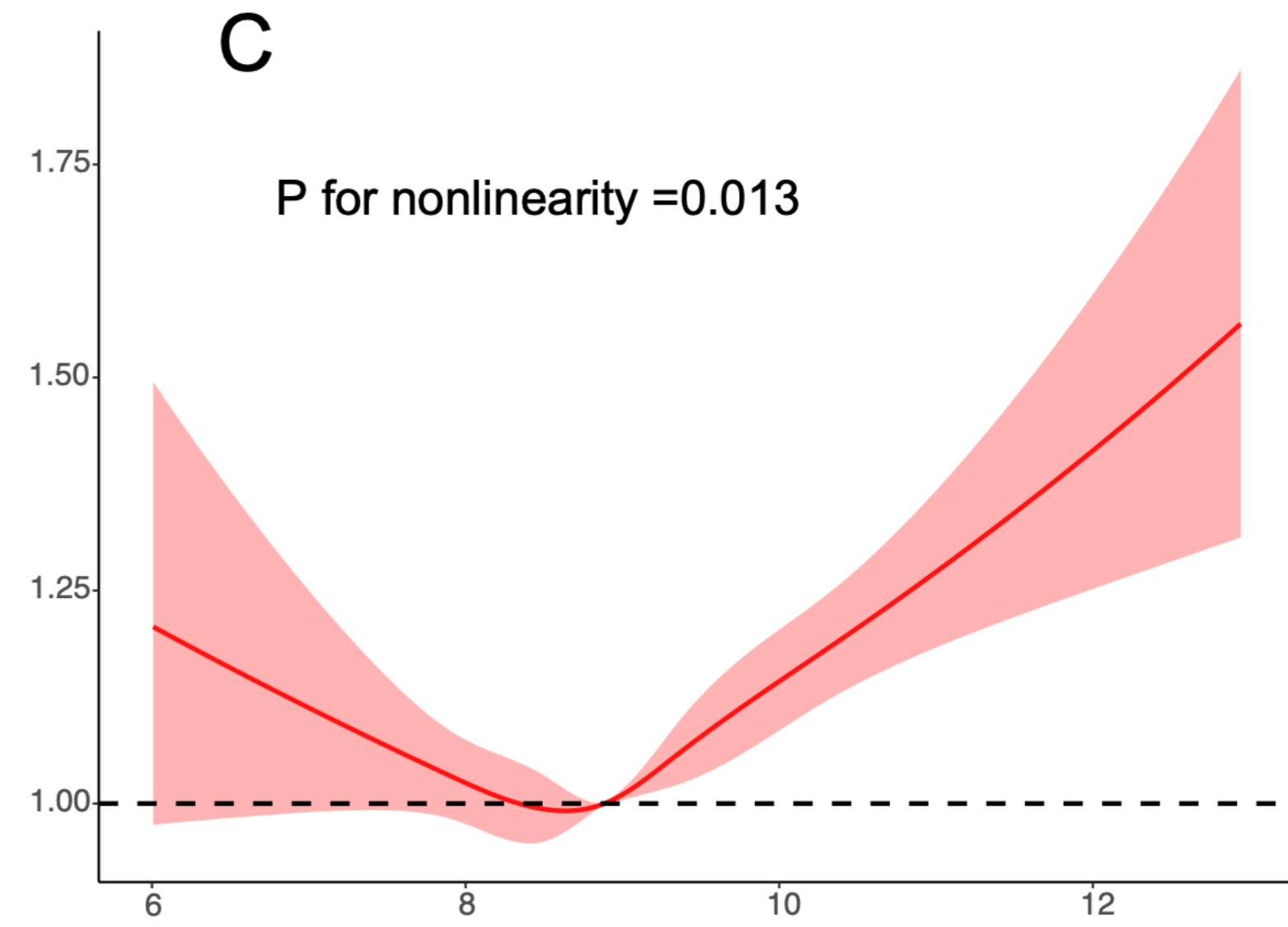

TyG

Supplement: Supplementary file 1 [file Image_1.pdf]
