# Supplementary material for: Prognostic value of the triglyceride-glucose (TyG) index for renal function progression in patients with CKD stages 3–4
Source: Front Nutr. 2026 Apr 28;13:1744275. doi: 10.3389/fnut.2026.1744275 (PMC13161084; doi:10.3389/fnut.2026.1744275)
Supplement: Supplementary file 2 [file Table_1.docx]

**Supplementary Table 1. The association Between TyG Index and renal progressionin CKD Stages 3–4: Complete-Case Analysis vs. Multiple Imputation**

| **Exposure** | **Reference** | **Dataset** | **n** | **Events** | **HR (95% CI) Q1** | **HR (95% CI) Q3** | **HR (95% CI) Q4** | **HR (95% CI) per 1-unit TyG** | **P for trend** |
| --- | --- | --- | --- | --- | --- | --- | --- | --- | --- |
| TyG quartiles | Q2 | Complete case | **47,950** | **17,545** | 1.02 (0.98–1.06) | 1.03 (0.99–1.08) | 1.16 (1.11–1.22) | — | <0.001 |
| TyG quartiles | Q2 | Multiple imputation | 53,607 | 19,619 | 1.01 (0.97–1.05) | 1.02 (0.98–1.07) | 1.17 (1.12–1.22) | — | <0.001 |
| TyG (continuous) | — | Complete case | **47,950** | **17,545** | — | — | — | 1.09 (1.06–1.12) | <0.001 |
| TyG (continuous) | — | Multiple imputation | 53,607 | 19,619 | — | — | — | 1.10 (1.07–1.12) | <0.001 |
