# Supplementary material for: Prognostic value of the triglyceride-glucose (TyG) index for renal function progression in patients with CKD stages 3–4
Source: Front Nutr. 2026 Apr 28;13:1744275. doi: 10.3389/fnut.2026.1744275 (PMC13161084; doi:10.3389/fnut.2026.1744275)
Supplement: Supplementary file 3 [file Table_2.docx]

**Supplementary Table 2. Association Between TyG Quartiles and Renal Progression Using Fine–Gray Competing Risk Model (All-Cause Death as Competing Event)**

| TyG Quartile | Subdistribution HR (95% CI) | P-value |
| --- | --- | --- |
| Q1 | 1.03 (0.99–1.07) | 0.12 |
| Q2 (ref) | 1.00 (reference) | — |
| Q3 | 1.02 (0.98–1.06) | 0.28 |
| Q4 | 1.16 (1.11–1.21) | <0.001 |

Adjusted as in Model 3, Q2 is the reference.
